# Context of water transport related drownings in Bangladesh: a qualitative study

**DOI:** 10.1186/s12889-019-7871-1

**Published:** 2019-11-27

**Authors:** Jagnoor Jagnoor, Caroline Lukaszyk, Kamran ul Baset, Rebecca Ivers, Shamima Easmin, Aminur Rahman

**Affiliations:** 1 The George Institute for Global Health, University of New South Wales, 311-312, Third Floor, Elegance Tower, Plot No. 8, Jasola District Centre, New Delhi, 110025 India; 2 The George Institute for Global Health, University of New South Wales, Sydney, New South Wales Australia; 3 Centre for Injury Prevention and Research, Bangladesh (CIPRB), Dhaka, Bangladesh; 4 School of Public Health and Medicine, Faculty of Medicine, UNSW Australia, Sydney, NSW Australia

## Abstract

**Background:**

Bangladesh has one of the highest drowning mortality rates in the world. The use of unregulated water transportation may contribute to this burden, with 38% of all passenger traffic occurring by water. The present study aims to identify provider and end user perception on water transport related drowning risk, and barriers and facilitators for improving water safety practices.

**Methods:**

A qualitative study was conducted in a riverine area of Bangladesh, the Barishal division. Data was collected through 18 in-depth interviews, two small group discussions and six observations in February–March 2016. Content analysis was conducted, guided by domains of Haddon’s matrix for injury prevention.

**Results:**

A range of unsafe behaviours, practices and conditions were identified at pre event, event and post event stages of water transport related drownings. It was also recognised it is not only the regulation of water transport but other factors such as occupational insecurities, poor access to rescue services and healthcare, migration and capacity for skill development among providers that contribute to unsafe water transport practices and drowning risk.

**Conclusion:**

There are some immediate measures that can be implemented, with some monitoring and accountability processes for water transport safety. However, there is need for robust data to quantify transport related drowning, making a case for prioritization and action by relevant stakeholder such as government and transport providers.

## Background

Drowning is the third leading cause of unintentional injury mortality worldwide [1] with over 90% of drowning deaths occurring in low- and middle-income countries (LMICs) [2]. A number of factors are associated with an increased risk of drowning including a lack of swimming ability, the use and access to open water bodies, and inadequate child supervision around water [2]. Travelling over water can increase exposure to drowning risk and as a result, stringent safety requirements exist for water transport vessels in many high-income countries (HICs). These include legislation surrounding boat licencing and registration, requirements for lifejackets and safety equipment on board vessels, enforcement of safe speeds and safe distances from shore, and the presence of protocols for boating emergencies [3].

Waterways are a common avenue of transport in many LMICs including India, South America, Vietnam and Cambodia [4]. In this context, water transport is significantly less regulated than in HICs, with informal transportation sector, competing priorities and limited resources [5]. As a result, daily commuting often takes place on overcrowded and unsafe vessels, operated by staff who have not been appropriately trained to recognize dangerous conditions or perform high-seas navigation [6], increasing risk of boat capsize and collision. Improving the safety of water transport systems in LMICs is associated with a number of challenges including competing priorities for limited resources and a lack of government commitment [6]. By identifying the major gaps in water transport system safety, priority can be given to the implementation of relevant and effective interventions addressing safety issues of primary concern.

Drowning rates in Bangladesh are some of the highest in the world (11.7/100,000 population), double the global average (under 5/100,000) and almost ten times higher than the rate in high-income countries like Australia (1.19/100,000) [7–9]. The use of unregulated water transportation may contribute to this burden, with 38% of all passenger traffic occurring by water [4]. Within Bangladesh, the incidence rate of ferry fatalities per kilometres travelled is comparable to that of road transport fatalities [10]. The Barishal is a riverine division in south–central Bangladesh. Fatal drowning rates are nearly three times higher in the Barishal Division than rest of the country [7].

The present study aims to identify perceptions of providers and end users on water transport related drowning risk, and barriers and facilitators for improving water safety practices in the riverine division of Barishal, Bangladesh. This information can be used to guide the formulation of policy and legislations, addressing the major gaps in water transport safety in Bangladesh.

## Methods

### Study design

A qualitative study was conducted via in-depth interviews (IDI), small group discussions (4 to 6 participants) and observations.

### Study context

The Barishal Division of Bangladesh is a highly riverine area with major rivers including Kirtankhola, Arial Khan, Khoyrabad, Kalijira and Sandha. The Barishal River Port is a major port, and water transport is the most common means of commute to the capital of Bangladesh, Dhaka, and other districts of the Barishal Division.

### Participants and data collection

Purposive sampling was used to select individuals who a) frequently used water transport (more than 3 trips a week), b) were operators of water transport vessels for public use or c) were owners of water transport vessels supplying the vessels for public use. Participants were only eligible for inclusion if they were aged 17 years or over and provided written informed consent prior to their involvement in the study. On average, interviews took 40–60 min, whilst group discussions took just over an hour. The epidemiological domains of injury prevention and control guided the development of broad themes for data collection tools, exploring barriers and facilitators for safe practices pre, during the event and post event (see Additional files 1 and 2 for data collection guides). Data collection tools were tested among study team members. Data were collected in locations acceptable to the interviewees. Interviews were conducted in Bengali, recorded, and translated and transcribed in English. Hand written field notes were also made by a note taker.

There were three trained data collectors (SE, MS and KS) who were oriented to the topic and data collection tools prior to data collection. To minimise data loss, interviews were jointly translated and transcribed by the interviewing researcher and the translator, and a member of the research team checked the translated transcripts. Interviews ceased when saturation of meaning was reached.

Observations by data collectors while travelling on a variety of water transport vessels in the study were conducted in the area, to capture the context of water transport use and prompt inductive analysis. Structured note taking, sketching and photography were used to document relevant safety practices and safety hazards observed during each trip. Observations were conducted over a 3 hour period. All data collection tools were designed to investigate the presence of risk factors jeopardising safety on water transport and the context of drowning during the use of water transport, Additional file 1. Unfavourably, field conditions did not allow for team briefings. Data was collected over 2 months from February to March 2016.

### Data analysis

Transcripts were imported into NVivo qualitative data analysis software (QSR International Pty Ltd. V.11, 2015). Content analysis was applied to the transcripts whereby overarching themes were initially developed. Thereafter, deductive thematic coding of the data was undertaken. NVivo was used to arrange the text according to codes and manage the codes in the interpretive phase. Three researchers (JJ, CL and DM) independently conducted the coding and arrived at consensus on the analyses. Results are reported in alignment with the domains of Haddon’s Matrix [11], highlighting injury risk factors present pre, during and post a drowning event attributable to the host (passengers and operators of water transport), vector (the water transport vessel) and the physical transportation environment. This manuscript is reported in line with the COREQ (Consolidated Criteria for Reporting Qualitative Research) statement, supporting transparency in reporting qualitative research.

### Ethical approval

Ethical clearance for this study was obtained from the University of Sydney, Australia (project number: 2016/606) and Ethical Review Committee – Centre for Injury Prevention and Research Bangladesh.

## Results

In total, 18 in-depth interviews were conducted with boat, ferry and launch operators/ owners, and users of water transport. Both males (*n* = 14) and females (*n* = 4) were sampled, with participants age ranging from 17 to 70 years of age. Two small group discussions were conducted with transport passengers, one with all female participants (*n* = 7) and one with all male participants (*n* = 6). One further group discussion was conducted with transport providers, comprised entirely of males (*n* = 6). Participants ranged from 28 to 50 years of age. All 37 participants that were approached to partake in the interviews and focus group discussions gave consent and participated. Six observations were conducted on a range of transport vessels including river trawlers, traditional boats, launches and ferries.

Details of the participants are summarised in Table 1.

**Table 1:** Demographic characteristics

#	Gender	Age bracket (years)	Transport user / provider	Occupation
In-depth interviews				
P1	F	17–20	User	Unknown
P2	F	17–20	User	Student
P3	F	30–40	User	Housewife
P4	M	40–50	User	Fishing business
P5	M	50–60	Provider	Fisherman, occasionally takes passengers
P6	M	40–50	Provider	Ferry driver
P7	M	60–70	User	Buys and sells fish
P8	M	40–50	Provider	Ferry operator
P9	M	Unknown	User	Works for a Climate Change Adaptation project
P10	M	30–40	Provider	Boatman
P11	M	30–40	Provider	Launch operator
P12	M	40–50	User	Farm and household work
P13	M	40–50	Provider	Boat owner, Fisherman
P14	M	50–60	Provider	Boating business (owns 5 boats)
P15	M	20–30	User	Senior bank officer
P16	M	70–80	User	Cultivator
P17	F	30–40	User	Housewife
P18	M	40–50	Provider	Launch clerk
#	Gender	Age range (years)	Number of participants	Transport user / provider
Small group discussions				
SGD1	F	Unknown	7	User
SGD2	M	28–50	6	Provider
SGD3	M	Unknown	6	User

For context, the qualitative data were collected alongside a large household survey in the population with the primary aim of determining drowning mortality rates and its risk factor in the Barishal population. The survey found that for adults aged 18 years and above, only 10% (*n* = 20,487/199,398 survey population) reported using a water transport in the last week and less than 1% of fatal or non-fatal drowning event was associated with transportation. The average number of trips reported were 3*. (manuscript under review).*

**Fig. 1:**
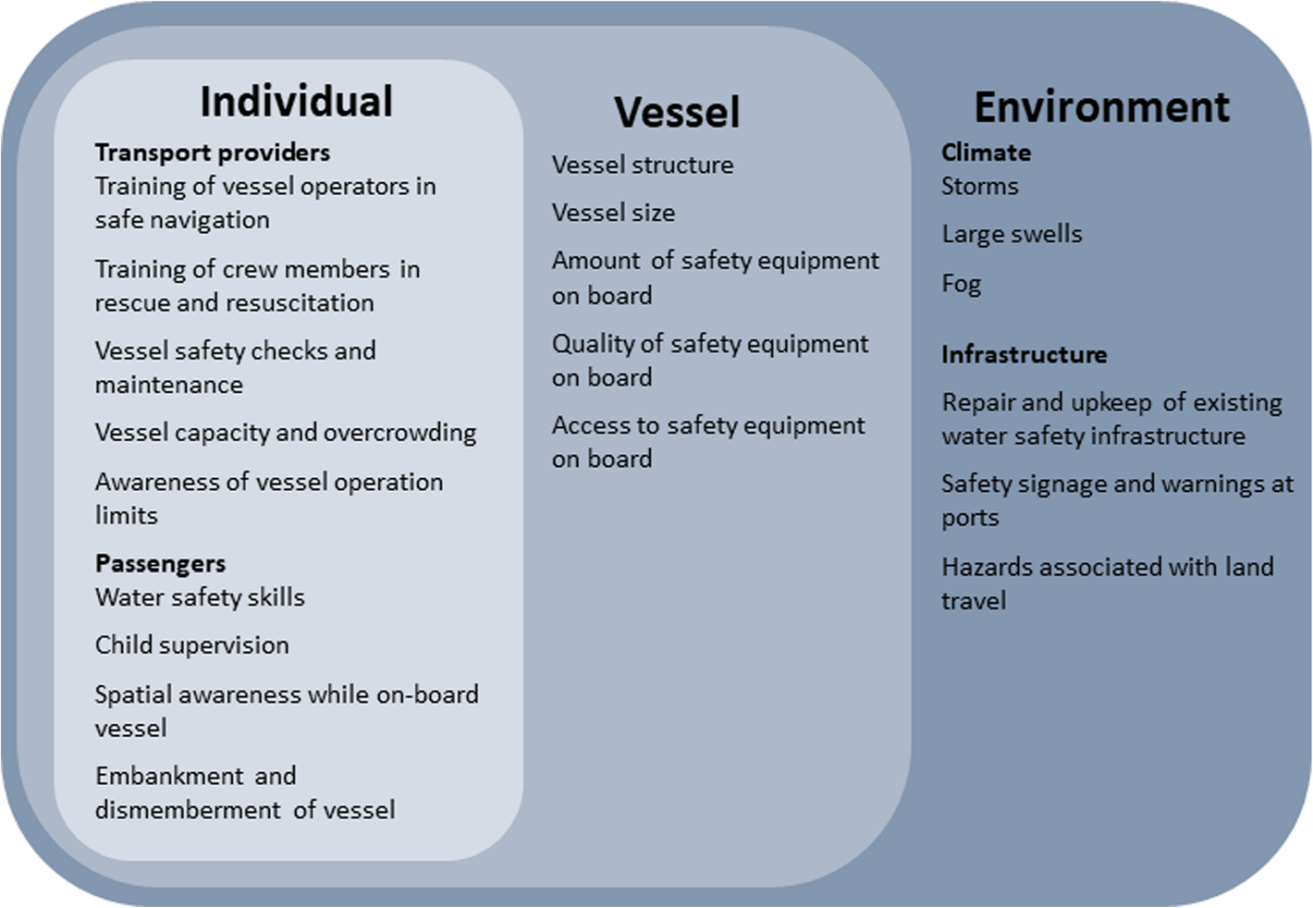
Factors that influence the safety of water transport in Bangladesh corresponding to the three domains of Haddon’s Matrix

Study results are grouped into three key areas: 1) Factors associated with individuals (Host) 2) Factors associated with water transport vessels and 3) Factors associated with the transport environment. Within each area, safety factors are attributed to pre, during and post drowning-event chronology, corresponding to Haddon’s Matrix, for injury prevention [11], identifying time points for opportune intervention to prevent drowning. Key themes are summarised in Fig. 1.

### Pre-event

#### Person (host)

Several vessel operators reported checking the weather forecast prior to travelling over water, with many suspending services if inclement weather was expected. Other vessel operators were unable to cancel services in these circumstances, requiring the income earnt through each trip to support themselves and their families.

*“When they [boat operators] are facing financial instability they go with their boats even after knowing that there is some risk. To feed themselves and their family their kids they go out for work. We [fishermen] are afraid but we still have to go.”*

(Fisherman, P5)

A smaller proportion of service providers were highly confident in their ability to navigate vessels through inclement weather including storms and fog, or were highly confident in their vessels’ structure to withstand volatile conditions.

**I:** *“Are there any problems in the ferry in the time of storm?”*

**R:** *“No, [there are no] problems. In there is a big engine and it is a big vehicle.”*

(Ferry operator, P6)

Overcrowding was reported to be common on transport vessels, particularly during holiday periods. Eid ul-Fitr and Eid ul-Adha is an important religious holiday celebrated by Muslims worldwide that marks the end of Ramadan. Transport providers reported passenger demand for water transport to increase three to four fold during Eid. In response, operators confess to permitting vessel overcrowding with boat carrying capacity not regulated by local authorities. Overcrowding vessels during holiday periods was considered a good opportunity to earn additional income for boat operators and owners.

*“During the Eid the pressure of passengers is high. We carry more passengers because everyone has to go home. Suppose in my launch I can take 300 passengers but during the Eid it is 500.”*

(Launch operator, P11)

Service providers placed primary responsibility for water transport safety on the passengers themselves, with passenger carelessness and lack of awareness thought to play major roles in water transport-related incidents. Signage promoting safe behaviour while using water transport was displayed in port areas, and staff working on-board vessels reported to verbally warn people to cease risky behaviour while travelling. Neither approach was considered to have impact on passengers. Passenger distraction was attributed to mobile phone use and a resulting lack of spatial awareness. Poor child supervision by parents was associated with children falling overboard, both during embarkment and disembarkment from the vessel and over the course of the trip, when climbing through gaps in vessel railings.

*“It happens because of the passengers faults. I was there during this incident. The ferry was tied [to the dock]. A kid was crossing the side with his father or uncle, the kid was behind him. All of a sudden, the kid fell down and he was not found instantly. The dead body was found later on. It happens because of the carelessness of the passengers.”*

(Ferry operator, P6)

Passengers were well aware of the transportation challenges of overcrowding during holiday periods, but often chose to travel during public holiday and festival times to mitigate income loss. Passengers were reported to jump from vessels onto the shore prior to docking, often resulting in other passengers being knocked into the water.

*“Sometimes passengers get down by jumping before the adjustment of the boat onto the shore. At that time, accident may happen. We want to keep them safe though people are hasty all the time to get down. Because of the hastiness, problems happen.”*

(Boatman, P10)

#### Water transport – vessel (vehicle)

Passengers attributed varying levels of risk to different types of water transport vessels. Ferries were considered the safest vessels due to their large size, stability on the water, and frequent safety checks and maintenance. Trawlers were reported to be old and neglected, many with rotted wooden decks and lacking on-board shelter causing passengers to be exposed to rain and wind during commute. While stable landing stations were available for ferries, smaller boats required passengers to used wooden ladders and bamboo poles to board and disembark.

*“There is a stair for the passengers and there is also bamboo kept beside the stair for the old people. They can get up by holding this bamboo.”*

(Launch operator, P11)

Water transport providers acknowledged the importance of conducting regular safety checks on their vessels and described undertaking maintenance and repairs in a timely manner despite associated costs.

*“I myself do [a safety check] each time I take the boat out, whether there is any problem in the boat or not. Those who are sailing the boat they themselves check their boats for faults. If there is anything found, we repair it.”*

(Fishermen, P5)

**Fig. 2:**
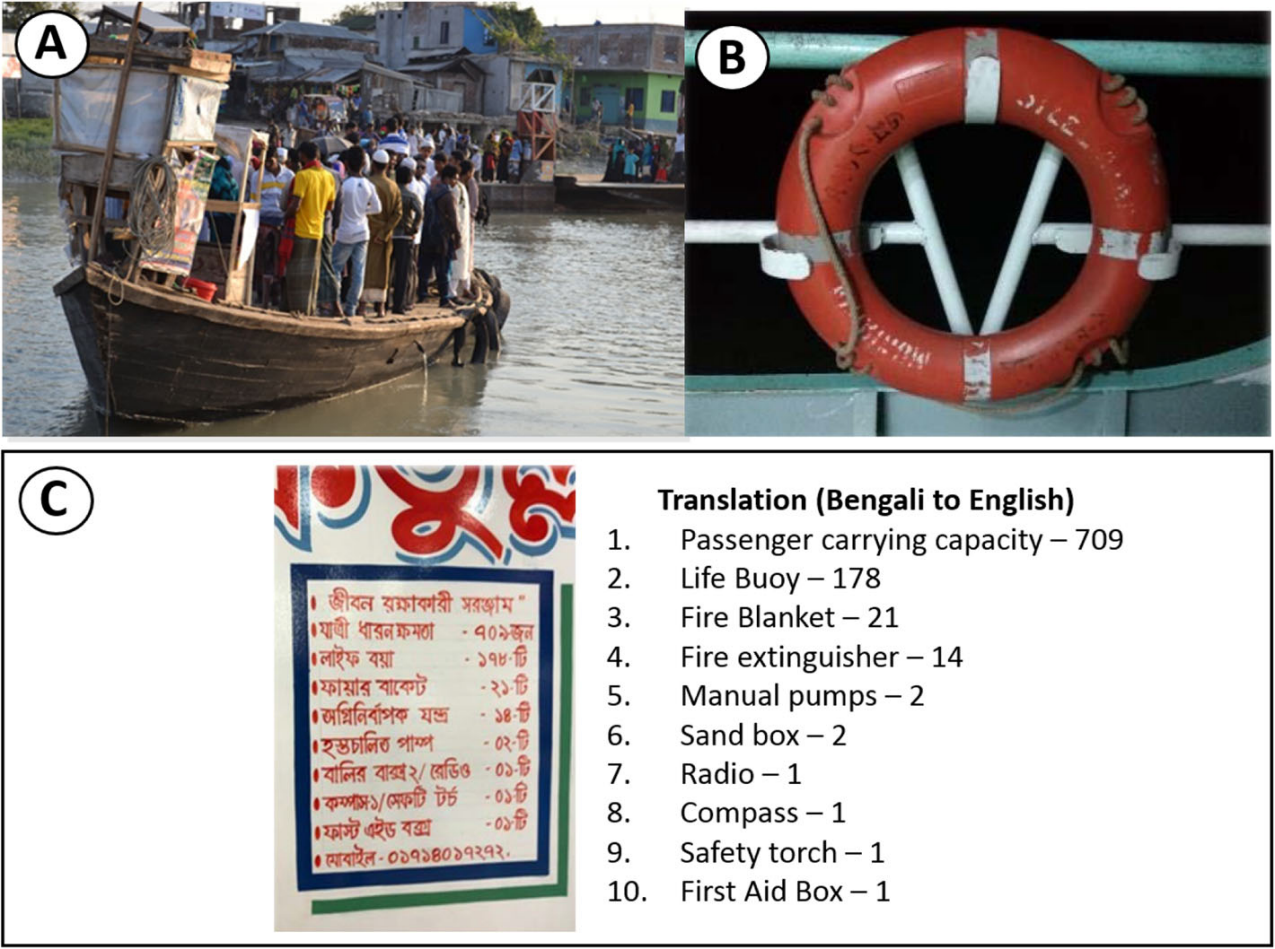
Photographs taken during water transport observations: **a** overcrowded traditional wooden boat (**b**) old and unmaintained life buoy on a large launch (**c**) safety equipment list observed on a large launch with English translation

Whilst vessel structural safety was ensured by owners, the availability and quality of safety equipment on-board vessels varied greatly. From the six observed vessels, three had no safety equipment on board. Of the three vessels carrying safety equipment, the number of flotation devices present did not correspond to the vessels’ carrying capacity. This is demonstrated in a safety equipment list on a large launch, which listed 178 life buoys on a trawler with a passenger carrying capacity of 709 people (Fig. 2). As seen in Fig. 2, there are no handrails, and the trawler is overcrowded.

Further, the quality of available floatation devices observed was poor, with many being old, damaged and unusable.

*“It is seen that, if we keep extra buoys and life jackets separately for general public or for the passengers and if these are left unused for a long period, then these become damaged.”*

(Ferry operator, P8)

In the past, NGOs were reported to distribute free safety equipment. However, this occurred inconsistently with not enough equipment provided to meet vessel carrying capacity.

#### Transport environment

Ferries were reported to run infrequently, with poor coordination of services between ports. As a result, participants were often required to use privately owned water transport vessels for some portions of their journey, such as speedboats. These were entirely unregulated and therefore presented varying levels of risk.

Seasons that bring high rainfall and frequent storms were considered dangerous periods for travel over water. Despite this, a number of water transport providers continued operating during these periods, largely due to financial requirements. The quote below reflects on the economic circumstances that drives risk-taking behaviour during bad weather.

*“When they [boat operators] are facing financial instability they go with their boats even after knowing that there is some risk. To feed themselves and their family their kids they go out for work. We [fishermen] are afraid but we still have to go.”*

(Fisherman, P5)

Passengers stated that water transport was a safer option during wet season compared to land travel. Frequent rain deteriorated the road conditions, significantly increasing the risk of road traffic crashes for both buses and private vehicles.

*“If there is rain then travelling with boat increases, because all the roads become muddy. More than ten people travel on my boat. Water increases during that time.”*

(Boat owner and fisherman, P13)

### During-event

#### Person (host)

Crew members and other passengers primarily performed the rescue of passengers who fell over-board water transport vessels. Crew members described land/dry rescue attempts of throwing ropes, bamboo sticks and other buoyant materials into the water to keep submerged people afloat. Entering the water to perform rescue was considered high risk, with a number of secondary drowning cases recalled where rescuers were pulled away from the vessel by tides or were pushed below the water by a panicked drowning person.

*“If I go in the water to save the drowned then I will also drown and die because they will grapple me so that they can come out from water. In that case I will not able to rescue them.”*

(Student, P2)

Passengers most vulnerable to drowning risk in this situation were identified as those who had limited swimming ability, with women, children and the older people included in this group. For women, swimming ability was thought to be hindered by long hair and traditional clothing (sari, hijab), which become heavy when wet.

*“Because normally, [women] wear more clothes. When these cloths get wet, they become heavy. How long they will be able to swim?”*

(Fishing business owner, P4)

#### Water transport – vessel (vehicle)

Safety equipment was often stored in areas that are not readily accessible, such as locked storerooms for fear of theft. This was identified as a barrier to performing timely rescue at the time of a drowning event. Similarly, there were reflections on boat capsizing due to overcrowding, capacity of the vessel.

*“Even if [buoys] exist on the launch, these are kept in a store room and would be of no use at the time of need.”*

(NGO worker, P9)

In the quote below, the participant emphasises on need to regulate number of occupants in vessels, and identifies overcrowding as a major risk to safe transportation.

*“I can eat rice one plate but if I try to eat one bowl so would it be good?” (SGD, 1)*

#### Transport environment

During wet season, both passengers and boat operators reported experiencing sudden weather changes and being caught at sea during unanticipated storms where boat engines ceased to operate due to large swells. Fog during the winter season led to poor visibility, preventing safe navigation of vessels. Passengers were reported to be thrown overboard due to large swells, with vessel capsize also reported during extreme conditions. Vessel capsize was more likely to occur with overcrowding.

*“I was going by this side from Dhaka and when I was near Padma River, it started to rain. Suddenly the storm broke out when we entered into the River. People started to cry. Water was coming into the boat and people were panicking more. But the shipman was driving straight without stopping anywhere. After a while it calmed down and everyone was relaxed. Even I was scared at that time.”*

(Buys and sells fish, P7)

### Post-event

#### Person (host)

Traditional resuscitation practices post-rescue were considered best practice, often implemented by crew members and other passengers. Common practices included pressing a persons’ stomach to induce vomiting or rubbing oil on their body to warm the skin.

*“If someone swallows water then people try to bring out that water from the stomach by pressing on belly.”*

(Boat owner and fisherman, P13)

Vessel operators recalled training previously provided by both national and international NGOs, focused on safe vessel operation and rescue skills. This training was considered to be delivered sporadically and in an inconsistent manner, limiting its effectiveness.

#### Water transport – vessel (vehicle)

There was a lack of operational and accessible floatation devices on-board, however data did not support any barriers or facilitators for rescue and resuscitation, which were dependent on the vessel.

#### Transport environment

Lack of organised rescue systems was perceived as a major barrier to preventing drowning deaths associated with water transport use. No rescue vessels were reported to be operating in the study area. As a result, rescue following capsize was dependant on the availability and responsiveness of other water vessels in close range. Data on deaths or water transport related incidents are often not reported to the police, as has little implications with largely unorganised water transport sector.

*“The trawler fell into a rainstorm and sank. Somehow, people got to another trawler by swimming. Those who failed to get on another trawler by swimming, they were flown away. Many such people died in sea.”*

(Transport user, P11)

### Suggestions to improving the safety of water transport

Enforcement of boating safety standards by government authorities was considered crucial to ensuring water transport vessels consistently meet safety requirements and are operated in a safe manner. Participants (operators and users) recommended that the government take responsibility for managing vessel registration, performing vessel safety checks and restricting wharf access in poor weather conditions.

*“If there is enforcement of law then it is possible. If you go to Kawrakandi you will see no one is allowed to get on speed boat without life jacket during rainy season. If this is introduced here, then they will use [lifejackets].”*

(NGO Worker, P9)

Safety equipment was considered to be unaffordable by a number of boat owners, who suggested government provide free or subsidised equipment as a solution. Boat owners additionally suggested that the government provide financial support for vessel maintenance to ensure all vessels are able to maintain structural safety.

*“[Buoys] are not available unless these are given by the government. I will not buy the buoys from the market. If I will buy them, then I will use those personally. I will buy for my own safety, if I travel by boat.”*

(Fishing business owner, P4)

Opportunities for training in rescue and resuscitation were requested by transport providers. This was thought possible through improved coordination between NGO and government initiatives. Additionally, the establishment of water accident response systems was considered necessary, particularly during high-risk travel periods, such as during holidays and wet season. Boat operators suggested more effective signage be displayed in ports promoting safe behaviour when using water transport.

Improvements in the coordination of water transport services was suggested by passengers for mitigating overcrowding. Suggestions were also made to improve road infrastructure to promote the use of land transport during wet season. The construction of bridges was considered an effective approach to decreasing reliance on water transport.

## Discussion

To our knowledge, this is the first study exploring transport provider and user perspectives on water transport related drowning risk in Bangladesh. By identifying barriers and facilitators for improving water safety practices in the riverine division of Barishal, it provides an important foundation from which to develop future interventions and policy. Drowning deaths occurring from water transport vessels are greatly under-reported globally, as they are often classified as transport accidents rather than drowning deaths [12]. This issue is likely exacerbated in LMICs with poorer monitoring structures in place. To bring greater focus onto the issue of water transport safety in Bangladesh, this study identified a number of high-risk practices associated with the use of water transport in the Barishal Division of Bangladesh and the contributing factors. A combination of unsafe passenger and water transport provider behaviours, hazardous transport vessels, volatile climate and under-resourced transport infrastructure contributed to an overall unsafe water transport system. Participants provided suggestions for improving water transport safety, with the vast majority of recommendations requiring increased government commitment and resource investment. These solutions are reflective of safe systems approaches which advocate for the development of environments that reduce the risks of injury. An example is the Save LIVES package for road injuries, which may be adapted for water-transport injuries and include strategies such as water vessel safety standards, enforcement of water transport laws, effective rescue response and leadership on water transport safety [13].

In 2014, the World Health Organization (WHO) released the first Global Report on Drowning which included evidence-based recommendations for improving the safety of water transport [2]. The report recommends countries to set and enforce safe boating, shipping and ferry regulations, providing International Maritime Organization shipping standards [12] as a reference for the design, equipment and operation requirements of water transport vessels. Recommendations for ferries include appropriate numbers of personal floatation devices on board, regular vessel maintenance, appropriate training provided to captain and crew, established and rehearsed evacuation plans, adherence to appropriate travel routes, enforcement of passenger capacity and restricting travel in inclement weather [2]. Recommendations for small boats include requiring personal floatation devices to be worn at all times by all passengers, and ensuring vessels contain a communication device, anchor, waterproof torch and set of paddles or oars [2]. This study looked beyond the WHO recommendations, adding to the literature what leads passengers to make ‘unsafe’ choices and what causes transport providers to poorly implement or ignore the regulations despite often being aware that risk is involved. For example, Article 55 of the Inland Shipping Ordinance prohibits voyage during a storm signal [13] (carrying penalties of up to 3 years imprisonment and fines) however, transport providers reported that they often went out in a storm regardless. Key drivers behind these choices included a lack of enforcement combined with livelihood needs. A lack of suitable alternatives for passengers combined with financial pressures explained why they also took risks on water transport. This knowledge of the decision making priorities and the environment in which these choices are made can meaningfully contribute to forming effective interventions in the future.

This study highlighted that a number of challenges exist in ensuring the implementation of these recommendations in LMICs, such as Bangladesh. This is primarily due to substantial resource requirements and a need for strong, committed governance. Competing health and economic priorities cause transport-related drowning to not be a primary concern of policy makers, transport providers or often passengers. There is lack of robust quantitative data on transport related drowning, to enable government action.

Once water transport safety is acknowledged as a problem of concern, multisectoral collaboration is a promising approach to ensuring effective governance is established for improving Bangladesh’s water transport system. This collaboration should involve representatives from various government departments and ministries (Ministry of Health and Family Welfare, Ministry of Labour and Employment, Ministry of Shipping, Ministry of Road Transport and Bridges, Ministry of Water Resources), multilateral and bilateral organisations, and representatives from relevant local non-government organisations. This type of collaboration can remove burden from central government and allocate tasks to different agencies corresponding to their core business, influence and available resources.

A major limitation of the study is that we were unable to include any government representative as regulators, or operators for example staff of Bangladesh Inland Water Transport Authority. Inclusion of wider group of stakeholders would have provided rich data on barriers form implementation and policy perspective. Secondly, the data was transcribed in Bangla and a licensed translator was used for English translation. However, as is with qualitative data some nuances could be lost, as the data collectors did not translate the data. In contrast a major strength of the study is that three researchers independently identified the themes, and two of them were part of the data collection team.

A comprehensive, evidence-based program addressing the major gaps in water transport safety must consider risk factors for drowning pre, during and post event. These strategies may be adapted from others implemented around the world. A promising intervention addressing pre-event risk involves the placement of government safety officers at ports to inspect the condition of transport vessels, inspect availability of quality and accessible safety equipment, ensure passenger capacity is maintained and enforce travel bans in inclement weather. This strategy has been employed by the Marine Transportation Office in busy ports of Thailand, although its efficacy has not been tested [14]. Authority to fine both transport providers and passengers for unsafe behaviour is a key driver of enforcement and sustainability of the position through the revenue raised. For example, enforcement of water safety law is a key component of Australia’s Maritime Safety Authority’s mandate, a branch of the police force known as the ‘Water Police’. Adapted strategies will need to be tailored for the Bangladeshi cultural context, such as excluding components that seek to control alcohol consumption around water as rates of alcohol use are low in this population [15].

To improve safety at the time of event, transport providers would benefit from training on safe and effective approaches to rescue and resuscitation, as is compulsory for all commercial boat operators in Australia. Additionally, establishment of official maritime search and rescue systems can enhance current response systems, improving safety post-event. Maritime rescue systems commonly comprise of lifeboats deployed at the coast with trained rescue staff and equipment in response to an accident at sea [14]. Each suggested intervention requires significant support and resource contribution from government and other relevant high-level stakeholders for effective, regulated and sustained implementation.

## Conclusion

A number of modifiable factors to prevent drowning have been identified through this study, with suggestions provided to improve the safety of existing water transport systems. A vast majority of the actions required for safe water transport practices require the involvement of government and non-government organisations, involving representatives from these stakeholder groups in this research would have contributed to study findings. Future research therefore, should focus on systems and seek input from a wider range of stakeholders. The findings of this research are anticipated to be relevant to other similar settings beyond Bangladesh where a high proportion of transport occurs over water.

## Supplementary information

**Additional file 1.** Mirco-level focus group guide for transport providers. Mirco-level focus group guide for transport users.

**Additional file 2.** Micro Level In-depth Interview Guide for Transport Users. Micro Level In-depth Interview Guide for Transport Providers.

## Data Availability

Requests for access to study data should be addressed to the corresponding author. Data will be made available to the scientific community with as few restrictions as feasible, while retaining exclusive use until the publication of major outputs.

## Abbreviations

COREQ: Consolidated Criteria for Reporting Qualitative Research
HICs: High-income countries
IDI: In-depth Interview
LMICs: Low-and middle-income countries
NGO: Non-government organisation
SGD: Small Group Discussion
WHO: World Health Organization
