# Supplementary material for: Context of water transport related drownings in Bangladesh: a qualitative study
Source: BMC Public Health. 2019 Nov 27;19:1567. doi: 10.1186/s12889-019-7871-1 (PMC6880553; doi:10.1186/s12889-019-7871-1)
Supplement: Supplementary file 1 — Additional file 1. Mirco-level focus group guide for transport providers. Mirco-level focus group guide for transport users. [file 12889_2019_7871_MOESM1_ESM.zip › FGD_Transport proivders_Micro_10 August 2016R3.docx]

**Micro-level focus group discussion for transport providers**

**Methods:** FGD

**Target Population:** Providers of water transport

| **Comprehensive Drowning Reduction strategy Barisal**  **FGD IDNO** \|__\|__\|__\|__\| **Facilitator Initials** \|__\|__\|__\| **Note-taker Initials** \|__\|__\|__\|  **Audio file**: \|__\|__\|__\|  **Community number:** \|__\|__\| **Date** \|__\|__/__\|__/__\|__\|  **Introduction**  I am ______________________________ from Centre for Injure Prevention and Research Bangladesh (Facilitator)  I am ______________________________ from Centre for Injure Prevention and Research Bangladesh (note-taker)   - Ask group to introduce themselves using first names - Capture demographic details – using first name for discussion - Explain general purpose of the study: - *For FGD:* To understand overall drowning situation and possible the reduction strategy - Aims of the discussion and expected duration (1 hour) - Who is involved in the process (other participants) - Why the participants’ cooperation is important - What will happen with the collected information and how the participant/target group will benefit - Ask group to define their own ground rules, for example: - Only one person talks at a time. - It is important for us to hear everyone’s ideas and opinions. There are no right or wrong answers to questions – just ideas, experiences and opinions, which are all valuable. - It is important for us to hear all sides of an issue – the positive and the negative. - Confidentiality is assured. “What is shared in the room stays in the room.” - Any questions? - Check position and functioning of tape recorder - Check for everyone’s consent to participate and be recorded - Refreshments will be served after the discussion   **Now I am going to introduce some topics one at a time about your experiences, and I hope you can discuss them together.** | |
| --- | --- |
| **Domain** | **Topic and Probes** |
|  |  |
| **Closing**  We are now approaching the end of our discussion. Is there anything else anyone would like to add that we have not talked about?   - Summarise - Thank participants - Provide extra information and contacts to participants   Collect participant demographic details | |

**Micro-level focus group discussion for transport providers**

| Topics to address in this FGD guide:   - Investigate transport provider perceptions of drowning risk and water safety - Explore current safe and unsafe practices of transport providers - Explore health seeking behaviours of transport related drowning events |
| --- |

| **Introduction** | |
| --- | --- |
| **Introductory statement**  Welcome, and thank you for agreeing to be part of today’s focus group discussion. We appreciate your willingness to participate and the time you have taken out of your day to take part.  My name is……………….and I am from the Centre for Injury Prevention and Research, Bangladesh in Dhaka. We are conducting these discussions with water transport providers in the Barisal community to understand your views and experiences in relation to transport-related drowning so that we can find out the best way to reduce it in Bangladesh and in your communities. We would like to find out from you what your thoughts/opinions and concerns are about drowning and how we can work together with you to reduce drowning related deaths and disabilities in your communities.  First I would like to cover some general rules about how this discussion will run:   1. We would like you all to do the talking and to hear from everyone in the group. 2. Only one person should speak at a time so please wait for the person talking to finish. 3. There are no right or wrong answers – every person’s opinion and view is important and we would like to hear a range of different views. 4. Anything discussed in this room remains in this room. We want everyone to feel comfortable about discussing any sensitive issue. 5. We will be recording these discussions because we want to capture everything you have to say accurately. But please be assured that we do not identify anyone by name in any report and your identity will remain anonymous. 6. Does anyone have any questions? | |
| **Explore participant background information** | Now I would like everyone to introduce themselves. Can you please tell us your name, age, occupation if any, and for how long you have been providing water transportation? |

| **Theme** | **Discussion point** | **Probes/clarifications** |
| --- | --- | --- |
| **Investigate transport user perceptions of drowning risk and water safety** | | |
| Awareness/Perception - general | - **What concerns do you have about safety of the water transport you provide?** (open to anything like time, safety, crowding, goods safety) - **What about drowning? Do you think there is a risk of passengers drowning when using water transport?** | - Do you feel the routes you regularly operate are safe? - Do you feel the vessels you regularly operate are safe? - What are your safety concerns when passengers and goods are moving on/off board? - Is it common for passengers to need rescue embarking, disembarking or during travel, and to be pulled out of the water? |
| **Explore current safe and unsafe practices of transport users** | | |
| Risk behaviours | - **What are some of the reasons you think passengers drown while using water transport?** | - What situations/conditions can increase the risk of passengers drowning or nearly drowning while using water transport? - Who do you think is most vulnerable to drowning while using water transport? Why is that? |
| Current practices | - **How do you ensure the safety of your passengers?** | - Are the vessels you operate often over-crowded? Do you ever deny passengers entry onto a crowded vessel? - Are you concerned about the weather conditions? Have you previously provided water transport during bad weather? What happened? - Does operations change at any time – during festival season, bad weather, holidays? If so – how? |
| Barriers | - **What are the barriers to providing safe water transport?** | - What are your main barriers ensuring the vessel you operate is safe? How could these barriers be minimised? |
| **Explore health seeking behaviours following transport related drowning events** | | |
| Experience of drowning events | - **Do you know of anyone who has had a drowning/near drowning experience whilst using water transport?** **Please describe the situation. What happened? How and when did this happen?** | - Did anyone attempt to help the person? If yes, who and how? - How do you think this could have been prevented? |
| During drowning event | - **If you saw a person fall overboard your vessel, into the water, what would you do?** | - Why would you take this action? - Would you try to save them yourself? Why is that? |
| Post drowning event | - **Do you know what the usual immediate practices are after a person is recovered from water?** | - Who would be responsible for administering these practices in your situation? - Would you be confident in administering them? |
| **Explore safety practices to prevent transport related drowning events** | | |
|  | - **What can be done to reduce injury/drowning related to water transport use?** | - Do you have any suggestions or ideas on how the water transport you operate could be made safer? - What sort of programs, resources or facilities would you need for this? - Who do you think are best placed for implementing and overseeing this? |
|  | - **Are there currently any measures that you take to keep passengers safer when they use the transport you provide?** | - What do you ensure to carry with you on the vessel? Is there any safety equipment on the vessel (rope, radio, life jackets and like)? - Have you seen or heard about any safety resources or programs? What do you think of those? - Have you seen or received any material in relation to these? |
| **Exit question** | | |
| During our conversation, W, X, Y, Z measures were mentioned that could be taken to reduce drowning in transportation. If you were to rank these from 1 to 5 (1 being most helpful and 5 least helpful for your household/community), what would the order be?  Does anyone have any other questions or anything you would like to say or discuss further in this group? | | |
