# Supplementary material for: Context of water transport related drownings in Bangladesh: a qualitative study
Source: BMC Public Health. 2019 Nov 27;19:1567. doi: 10.1186/s12889-019-7871-1 (PMC6880553; doi:10.1186/s12889-019-7871-1)
Supplement: Supplementary file 2 — Additional file 2. Micro Level In-depth Interview Guide for Transport Users. Micro Level In-depth Interview Guide for Transport Providers. [file 12889_2019_7871_MOESM2_ESM.zip › IDI_Micro_Transport Users_10AugustR3.docx]

**Micro Level In-depth Interview Guide for Transport Users**

Topics to address in this IDI guide:

- Investigate transport user perceptions of drowning risk and water safety
- Explore current practices taken by transport users
- Explore health seeking behaviours of transport related drowning events

| **Demographic questions** | | |
| --- | --- | --- |
| 1. | Gender of transport user (circle as appropriate): Male Female | |
| 2. | Age of transport user (in years): |  |
| 3. | Occupation of transport user: |  |
| 4. | Types of water transport regularly used: (passenger launches, passenger trawlers, passenger steamers, ferries, engine boats, country boats) |  |
| 5. | Average time spent per week using water transport (in hours): | |

| **Themes** | **Queries** | **Probe/ clarifications/remarks** |
| --- | --- | --- |
| **Introduction** | | |
| **Background information** | Explore participant background information | -Can you please tell me your name?  -Where are you travelling from and what is your destination? Why are you travelling? How long will you be travelling on water today (total number of hours)? |
| **Explore current practices taken by transport users** | | |
|  |  | -What are some common water transport routes that you use? How frequently do you use these?  -Do you use water transport for long distances? If **yes**, why and how frequently?  -Do you know who owns the water transport that you use? Do you know who operates the vessels?  -How much do you pay per trip, on average? |
| **Investigate transport user perceptions of drowning risk and water safety** | | |
|  | Current risk taking behaviours of transport users | -Do you think the regular water transport routes you use are safe? If **yes** or **no**, then why?  -Have you previously used water transport during bad weather (low fog, storms, heavy rain) and felt unsafe? If **yes**, please describe what happened.  -Have you ever been affected by water transport being cancelled due to bad weather? If **yes**, what was weather like? Do you know who chose to cancel it? Did you have an alternative method of transport that you could use?  -Do you think the water transport vessels you regularly use are safe? If **yes** or **no**, then why?  -Do you feel safe boarding transport vessels? Do you feel safe disembarking transport vessels? If **no**, then why? Is there someone who can provide extra assistance if you require it?  -Are there safer alternatives to the water transport you usually use? If **yes**, what are they and why don’t you use them? Why do you think they are safer?  -Is the water transport you use often over-crowded?  - *(If applicable)* Can you recall if there is any safety equipment on the vessels you regularly use? If **yes**, what is it? Do you think the equipment is usable? Would you know how to use it if you needed it? Would there be enough to help all of the passengers on the boat?  -If you were to fall overboard a vessel during a trip, would you be able to keep yourself afloat?  -Overall, do you think that the local water transport system is safe? If **yes** or **no**, then why?  - Is the operation/operator behaviour a concerns? (risk taking) |
|  | Identify possible safer route and system in terms of drowning. | -What do you think could be done to make water transport safer? Would the water routes need to be changed? What sort of facilities, staff and/or equipment could help from your perspective?  -Can you anticipate any barriers to implementing these changes and maintaining them over time? If **yes**, any thoughts how could these be overcome? |
| **Explore health seeking behaviours of transport related drowning events** | | |
|  |  | -Have you had any drowning/near drowning experiences whilst travelling? If **yes,** please describe.  -Is it common for passengers to need rescue and to be pulled out of the water while embarking, disembarking or during travel? If you know of any incidents, please share them.  -Have you ever assisted a passenger who has fallen overboard the vessel? If **yes**, please provide details.  -How are transport users usually rescued in drowning situations?  -Who are the people involved in the rescue and what are the associated risks for them?  -Once rescued, what are the usual immediate practices after recovery from water? |
| **Exit Question** | | |
| During our conversation, you mentioned W, X, Y, Z measures that could be taken to improve water safety for transport users. If you were to rank these from 1 to 5 (1 being most helpful and 5 least helpful for your situation), what would the order be? Why have you chosen this order? | | |
