# Supplementary material for: Context of water transport related drownings in Bangladesh: a qualitative study
Source: BMC Public Health. 2019 Nov 27;19:1567. doi: 10.1186/s12889-019-7871-1 (PMC6880553; doi:10.1186/s12889-019-7871-1)
Supplement: Supplementary file 2 — Additional file 2. Micro Level In-depth Interview Guide for Transport Users. Micro Level In-depth Interview Guide for Transport Providers. [file 12889_2019_7871_MOESM2_ESM.zip › IDI_Micro_Transport Providers_10AugustR3.docx]

**Micro Level In-depth Interview Guide for Transport Providers**

Topics to address in this IDI guide:

- Investigate transport provider’s perceptions of drowning risk and water safety
- Explore current practices taken by transport providers
- Explore health seeking behaviours of transport related drowning events

| **Demographic questions** | | |
| --- | --- | --- |
| 1. | Gender of transport provider (please circle): Male Female | |
| 2. | Age of transport provider (in years): | |
| 3. | Number of years spent working as a transport provider: |  |
| 4. | Types of vessels regularly operated (cargo ships, passenger launches, passenger trawlers, passenger steamers, cargo trawlers, ferries, engine boats, country boats): |  |
| 5. | Average time spent working as a water transport provider per week (in hours): |  |

| **Themes** | **Queries** | **Probe/ clarifications/remarks** |
| --- | --- | --- |
| **Introduction** | | |
| **Background information** | Explore participant background information | -Can you please tell me your name?  -What water routes do you frequently operate? |
| **Water Transportation Operation** |  | -Do you have a maximum passenger capacity for your vessel? If **yes**, do you often reach this? Do you turn passengers away if capacity is reached?  -How do passengers embark/disembark the vessel? How do less able passengers (children, elderly, disabled) embark/disembark the vessel? What happens if they need assistance?  -Do any other staff regularly work on the vessel? If **yes**, how many? What are their jobs and responsibilities? How do they learn the needed skills?  -*(where applicable)* Have you ever been provided with information on Bangladesh water transport regulations? If **yes**, can you please tell me about it? |
| **Investigate transport provider’s perception of drowning risk and water safety** | | |
| **Practices** | The routes | -Do you find the transport routes you regularly use safe? If **yes** or **no**, then why? ( season , time of the day) |
|  | The vessel | -Do you think that the vessel you operate is safe? If **yes** or **no**, then why?  -What do you ensure to carry with you on the vessel? Is there any safety equipment on the vessel (rope, radio, life jackets and like)? If **yes**, what equipment is it? Is it usable? Who provided it? Do you know how to use it? Would there be enough for every person if the vessel was at maximum capacity?  -Do you yourself or anyone inspect the vessels condition for safety?  -What do you usually do to maintain the condition of the vessel?  -What are your main barriers ensuring the vessel you operate is safe? How could these barriers be minimised?  -What type of resources or facilities do you require to make the vessel you operate safer? What can be done to ensure you have them – what would help? |
|  | Operations | -Have you previously provided water transport during bad weather (low fog, storms, heavy rain, cyclone)? If **yes**, please describe what happened. Do you think the safety of the passengers was put at risk?  - Are there a minimum number of trips you need to make each day? Is time a constraint? Do you engage in unsafe/high risk behaviour making up for time or money?  -Have you previously encountered fishing nets, bamboo, low water depth, live electrical wire or any other obstructions during a trip? If **yes**, please describe what happened. Do you think the safety of the passengers was put at risk? |
|  | The staff | -Are there usually any staff present at the shore, to assist with docking and embarking/disembarking passengers?  -How would you deal with a situation where passengers need rescuing in drowning situations?  -Have you ever attended any safety awareness programs from the transport authority or any other organisation? If **yes**, please describe. |
| **Explore health seeking behaviours of transport related drowning events** | | |
|  | Explore drowning and near-drowning experiences of water transport users | -What do you think the main reasons are for people drowning while travelling by water?  -Have you ever personally assisted a passenger who has fallen overboard the vessel? If **yes**, please provide details.  -Have you ever personally assisted a passenger who has fallen into water whilst getting on/off the vessel? If **yes**, please provide details.  -Is it common for passengers to need rescue and to be pulled out of the water?  -Do you know of people who have drowned while travelling over water? If **yes**, please provide details. |
|  | Explore practices after drowning event | -Once rescued, what are the usual immediate practices after recovery from water? |
| **Exit Question** | | |
| During our conversation, you mentioned W, X, Y, Z measures that could be taken to improve water safety for both transport users and transport providers. If you were to rank these from 1 to 5 (1 being most helpful and 5 least helpful for your situation), what would the order be? Why have you chosen this order? And why? | | |
